# 5′′-(2,4-Dichloro­benzyl­idene)-1′-(2,4-dichloro­phen­yl)-1′′-methyl-1′,2′,3′,5′,6′,7′,8′,8a’-octa­hydro­dispiro­[acenaphthyl­ene-1,3′-indolizine-2′,3′′-piperidine]-2,4′′(1*H*)-dione

**DOI:** 10.1107/S1600536812037956

**Published:** 2012-09-08

**Authors:** J. Suresh, R. Vishnupriya, R. Ranjith Kumar, S. Sivakumar, P. L. Nilantha Lakshman

**Affiliations:** a Department of Physics, The Madura College, Madurai 625 011, India; b Department of Organic Chemistry, School of Chemistry, Madurai Kamaraj University, Madurai 625 021, India; c Department of Food Science and Technology, University of Ruhuna, Mapalana, Kamburupitiya 81100, Sri Lanka

## Abstract

In the title compound, C_37_H_30_Cl_4_N_2_O_2_, the pyridinone ring adopts a twisted half-chair conformation. In the octa­hydro­indolizine fused-ring system, the piperidine ring is in a chair conformation and the pyrrole ring is twisted about the C—N bond linking the five- and six-membered rings. The mol­ecular structure features an intra­molecular C—H⋯O inter­action and the crystal packing is stabilized by C—H⋯π inter­actions.

## Related literature

For the importance of spiro compounds, see: Biava *et al.* (2006 ▶); Chande *et al.* (2005 ▶); Dandia *et al.* (2003 ▶); Shaharyar *et al.* (2006 ▶); Sriram *et al.* (2006 ▶). For related acenaphthyl­ene structures, see: Hazell & Hazell (1977 ▶); Hazell & Weigelt (1976 ▶); Jones *et al.* (1992 ▶); Sundar *et al.* (2002 ▶).
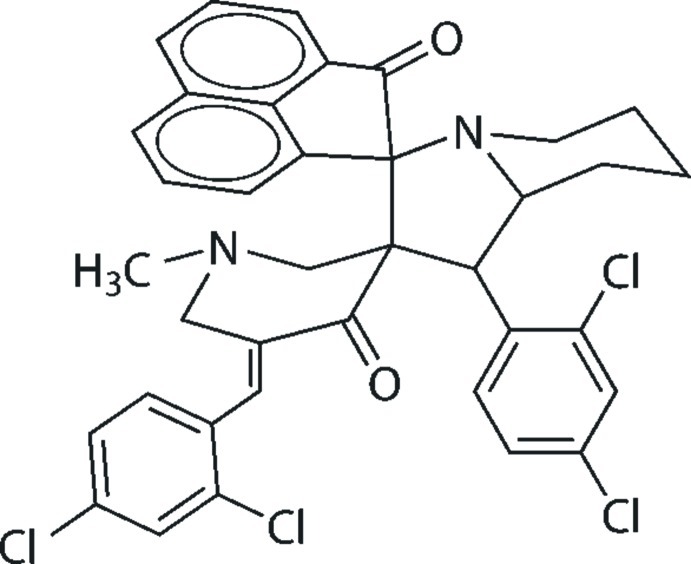


## Experimental

### 

#### Crystal data

C_37_H_30_Cl_4_N_2_O_2_
*M* *_r_* = 676.43
Monoclinic, 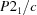

*a* = 8.5695 (2) Å
*b* = 16.1634 (5) Å
*c* = 23.8325 (7) Å
β = 92.399 (2)°
*V* = 3298.20 (16) Å^3^
*Z* = 4
Mo *K*α radiation
μ = 0.40 mm^−1^
*T* = 293 K
0.23 × 0.21 × 0.19 mm

#### Data collection

Bruker Kappa APEXII diffractometer
Absorption correction: multi-scan (*SADABS*; Sheldrick, 1996 ▶) *T*_min_ = 0.967, *T*_max_ = 0.974
29915 measured reflections
5750 independent reflections
4518 reflections with *I* > 2σ(*I*)
*R*_int_ = 0.029

#### Refinement

*R*[*F* ^2^ > 2σ(*F* ^2^)] = 0.041
*wR*(*F* ^2^) = 0.105
*S* = 1.08
5750 reflections
407 parameters
H-atom parameters constrained
Δρ_max_ = 0.60 e Å^−3^
Δρ_min_ = −0.55 e Å^−3^

### 

Data collection: *APEX2* (Bruker, 2004 ▶); cell refinement: *SAINT* (Bruker, 2004 ▶); data reduction: *SAINT*; program(s) used to solve structure: *SHELXS97* (Sheldrick, 2008 ▶); program(s) used to refine structure: *SHELXL97* (Sheldrick, 2008 ▶); molecular graphics: *PLATON* (Spek, 2009 ▶); software used to prepare material for publication: *SHELXL97*.

## Supplementary Material

Crystal structure: contains datablock(s) global, I. DOI: 10.1107/S1600536812037956/tk5147sup1.cif

Structure factors: contains datablock(s) I. DOI: 10.1107/S1600536812037956/tk5147Isup2.hkl

Additional supplementary materials:  crystallographic information; 3D view; checkCIF report

## Figures and Tables

**Table 1:** Hydrogen-bond geometry (Å, °) *Cg*1 is the centroid of the C32–C37 ring.

*D*—H⋯*A*	*D*—H	H⋯*A*	*D*⋯*A*	*D*—H⋯*A*
C8—H8⋯O2	0.98	2.50	3.122 (3)	121
C10—H10a⋯*Cg*1^i^	0.97	2.68	3.5969	158

Symmetry code: (i) 
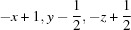
.

**Fig. 1.**
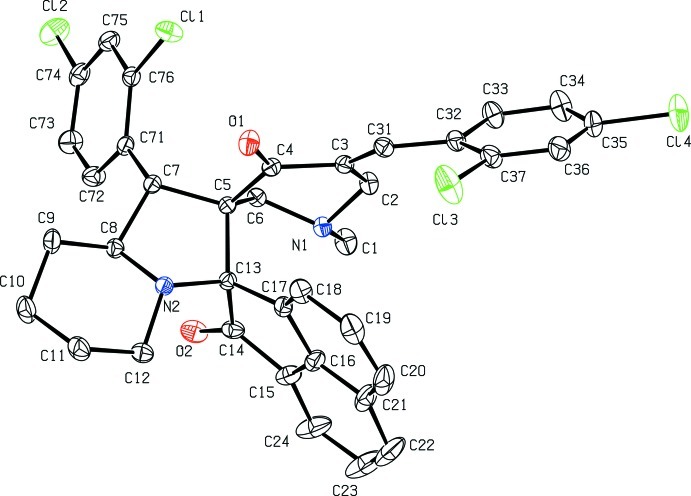
The molecular structure of (I), showing 20% probability displacement ellipsoids and the atom-numbering scheme. H-atoms are omitted for clarity.

## supplementary crystallographic information

### Comment

In general, spiro compounds and nitrogen heterocycles display good anti-mycobacterial activities (Chande *et al.*, 2005; Dandia *et al.*, 2003; Sriram *et al.*, 2006; Biava *et al.*, 2006; Shaharyar *et al.*, 2006) . It is also pertinent to note that the synthesis of biologically active indolizine derivatives continues to attract the attention of organic chemists, because of their wide spectrum of biological activity.

In the title compound (Fig. 1) the pyridinone ring adopts twisted half-chair conformation with atoms N1 and C5 deviating by -0.638 (2) and -0.465 (2) Å, respectively, from the least-squares plane defined by the other atoms (C2/C3/C4/C6). Within the octahydroindolizine fused ring system, the piperidine ring is in a chair conformation and the pyrrole ring is twisted about the N2—C8 bond. The C—C bond lengths and C—C—C angles in the acenaphthylene group compare with those of related structures (Hazell & Hazell, 1977; Hazell & Weigelt, 1976; Jones *et al.*, 1992; Sundar *et al.*, 2002). The observed conformation of the pyrrole ring may be due to the presence of an intramolecular C8—H8···O2 interaction (Table 1). The dihedral angle between the dichlorobenzene rings is 67.2 (1)° and these rings form angles of 46.8 (1) and 68.6 (1)° with the acenaphthene group, respectively. The sum of the bond angles at N2 of the pyrrole ring is 337.78°, indicating *sp*^3^-hybridization.

A weak C—H···π interaction (Table 1) *viz*., C10—H10···*Cg*1 is observed (*Cg*1 is the centroid of the ring C32—C37).

### Experimental

A mixture of 1-methyl-3,5-bis[(*E*)-2,4-dichloro phenylmethylidene]tetrahydro-4(1*H*)-pyridinone (1 mmol), acenaphthenequinone (1 mmol) and piperidine-2-carboxylic acid (1 mmol) was dissolved in isopropyl alcohol (15 ml) and heated to reflux for 60 min. After completion of the reaction as evident from TLC, the mixture was poured into water (50 ml), the precipitated solid was filtered and washed with water (100 ml) to obtain pure yellow solid. The product was recrystallized from ethyl acetate to obtain suitable crystals of (I) for X-ray analysis having a melting point of 480 K (Yield: 96%).

### Refinement

H atoms were placed at calculated positions and allowed to ride on their carrier atoms with C—H = 0.93–0.98 Å, and *U*_iso_ = 1.2*U*_eq_(C) for CH_2_ and CH H atoms and *U*_iso_ = 1.5*U*_eq_(C) for CH_3_ H atoms.

### Crystal data

**Table:**

C_37_H_30_Cl_4_N_2_O_2_	*F*(000) = 1400
*M**_r_* = 676.43	*D*_x_ = 1.362 Mg m^−^^3^
Monoclinic, *P*2_1_/*c*	Mo *K*α radiation, λ = 0.71073 Å
Hall symbol: -P 2ybc	Cell parameters from 2000 reflections
*a* = 8.5695 (2) Å	θ = 2–31°
*b* = 16.1634 (5) Å	µ = 0.40 mm^−^^1^
*c* = 23.8325 (7) Å	*T* = 293 K
β = 92.399 (2)°	Block, colourless
*V* = 3298.20 (16) Å^3^	0.23 × 0.21 × 0.19 mm
*Z* = 4	

### Data collection

**Table:**

Bruker Kappa APEXII diffractometer	5750 independent reflections
Radiation source: fine-focus sealed tube	4518 reflections with *I* > 2σ(*I*)
Graphite monochromator	*R*_int_ = 0.029
Detector resolution: 0 pixels mm^-1^	θ_max_ = 25.0°, θ_min_ = 2.1°
ω and φ scans	*h* = −10→10
Absorption correction: multi-scan (*SADABS*; Sheldrick, 1996)	*k* = −19→19
*T*_min_ = 0.967, *T*_max_ = 0.974	*l* = −28→28
29915 measured reflections	

### Refinement

**Table:**

Refinement on *F*^2^	Primary atom site location: structure-invariant direct methods
Least-squares matrix: full	Secondary atom site location: difference Fourier map
*R*[*F*^2^ > 2σ(*F*^2^)] = 0.041	Hydrogen site location: inferred from neighbouring sites
*wR*(*F*^2^) = 0.105	H-atom parameters constrained
*S* = 1.08	*w* = 1/[σ^2^(*F*_o_^2^) + (0.035*P*)^2^ + 2.1249*P*] where *P* = (*F*_o_^2^ + 2*F*_c_^2^)/3
5750 reflections	(Δ/σ)_max_ < 0.001
407 parameters	Δρ_max_ = 0.60 e Å^−^^3^
0 restraints	Δρ_min_ = −0.55 e Å^−^^3^

### Special details

**Table:**

Geometry. All e.s.d.'s (except the e.s.d. in the dihedral angle between two l.s. planes) are estimated using the full covariance matrix. The cell e.s.d.'s are taken into account individually in the estimation of e.s.d.'s in distances, angles and torsion angles; correlations between e.s.d.'s in cell parameters are only used when they are defined by crystal symmetry. An approximate (isotropic) treatment of cell e.s.d.'s is used for estimating e.s.d.'s involving l.s. planes.
Refinement. Refinement of *F*^2^ against ALL reflections. The weighted *R*-factor *wR* and goodness of fit *S* are based on *F*^2^, conventional *R*-factors *R* are based on *F*, with *F* set to zero for negative *F*^2^. The threshold expression of *F*^2^ > σ(*F*^2^) is used only for calculating *R*-factors(gt) *etc*. and is not relevant to the choice of reflections for refinement. *R*-factors based on *F*^2^ are statistically about twice as large as those based on *F*, and *R*- factors based on ALL data will be even larger.

### Fractional atomic coordinates and isotropic or equivalent isotropic displacement parameters (Å^2^)

**Table:**

	*x*	*y*	*z*	*U*_iso_*/*U*_eq_	
C1	−0.1592 (3)	0.47714 (17)	0.28069 (11)	0.0528 (6)	
H1A	−0.2056	0.4443	0.3091	0.079*	
H1B	−0.2226	0.4742	0.2466	0.079*	
H1C	−0.1518	0.5336	0.2930	0.079*	
C2	0.0685 (2)	0.49258 (14)	0.22637 (9)	0.0412 (5)	
H2A	0.0701	0.5507	0.2364	0.049*	
H2B	0.0059	0.4866	0.1917	0.049*	
C3	0.2331 (2)	0.46418 (12)	0.21678 (8)	0.0345 (5)	
C4	0.3245 (2)	0.42014 (13)	0.26274 (8)	0.0344 (5)	
C5	0.2333 (2)	0.38735 (12)	0.31178 (8)	0.0301 (4)	
C6	0.0974 (2)	0.44638 (12)	0.32082 (8)	0.0333 (4)	
H6A	0.0398	0.4287	0.3529	0.040*	
H6B	0.1364	0.5019	0.3280	0.040*	
C7	0.3420 (2)	0.37231 (12)	0.36537 (8)	0.0329 (4)	
H7	0.4501	0.3790	0.3542	0.039*	
C8	0.3193 (2)	0.28148 (13)	0.37979 (8)	0.0359 (5)	
H8	0.2270	0.2756	0.4024	0.043*	
C9	0.4565 (3)	0.23864 (15)	0.40912 (10)	0.0499 (6)	
H9A	0.4741	0.2615	0.4465	0.060*	
H9B	0.5499	0.2476	0.3883	0.060*	
C10	0.4239 (3)	0.14666 (16)	0.41318 (11)	0.0603 (7)	
H10A	0.5147	0.1188	0.4299	0.072*	
H10B	0.3370	0.1376	0.4372	0.072*	
C11	0.3854 (3)	0.11052 (15)	0.35563 (11)	0.0566 (7)	
H11A	0.3583	0.0526	0.3593	0.068*	
H11B	0.4763	0.1142	0.3328	0.068*	
C12	0.2503 (3)	0.15664 (13)	0.32705 (10)	0.0468 (6)	
H12A	0.2310	0.1354	0.2893	0.056*	
H12B	0.1565	0.1486	0.3478	0.056*	
C13	0.1744 (2)	0.29741 (12)	0.29436 (8)	0.0324 (4)	
C14	0.0011 (2)	0.28284 (13)	0.31192 (10)	0.0413 (5)	
C15	−0.0909 (3)	0.25445 (15)	0.26233 (12)	0.0519 (6)	
C16	0.0097 (3)	0.24771 (14)	0.21835 (10)	0.0490 (6)	
C17	0.1641 (3)	0.27180 (13)	0.23272 (9)	0.0387 (5)	
C18	0.2754 (3)	0.26134 (15)	0.19449 (10)	0.0523 (6)	
H18	0.3790	0.2750	0.2032	0.063*	
C19	0.2300 (4)	0.22907 (17)	0.14080 (11)	0.0714 (9)	
H19	0.3056	0.2220	0.1143	0.086*	
C20	0.0795 (5)	0.20812 (18)	0.12678 (12)	0.0804 (10)	
H20	0.0542	0.1881	0.0910	0.097*	
C21	−0.0373 (4)	0.21633 (17)	0.16545 (13)	0.0691 (9)	
C22	−0.1976 (5)	0.1935 (2)	0.15962 (18)	0.1010 (14)	
H22	−0.2364	0.1722	0.1256	0.121*	
C23	−0.2948 (4)	0.2021 (2)	0.2026 (2)	0.1132 (16)	
H23	−0.3991	0.1872	0.1969	0.136*	
C24	−0.2451 (3)	0.23215 (19)	0.25496 (17)	0.0831 (10)	
H24	−0.3138	0.2370	0.2840	0.100*	
C31	0.3044 (3)	0.47215 (13)	0.16838 (9)	0.0387 (5)	
H31	0.4048	0.4503	0.1681	0.046*	
C32	0.2482 (3)	0.51001 (14)	0.11587 (9)	0.0411 (5)	
C33	0.1588 (3)	0.58133 (16)	0.11353 (10)	0.0547 (6)	
H33	0.1286	0.6048	0.1470	0.066*	
C34	0.1126 (3)	0.61910 (19)	0.06375 (11)	0.0678 (8)	
H34	0.0530	0.6672	0.0636	0.081*	
C35	0.1566 (3)	0.58405 (18)	0.01424 (11)	0.0617 (7)	
C36	0.2467 (4)	0.51463 (17)	0.01395 (10)	0.0624 (7)	
H36	0.2769	0.4919	−0.0197	0.075*	
C37	0.2923 (3)	0.47867 (15)	0.06447 (10)	0.0516 (6)	
C71	0.3175 (2)	0.43159 (13)	0.41354 (8)	0.0352 (5)	
C72	0.2167 (3)	0.41435 (15)	0.45610 (9)	0.0479 (6)	
H72	0.1644	0.3639	0.4554	0.058*	
C73	0.1907 (3)	0.46867 (16)	0.49932 (10)	0.0528 (6)	
H73	0.1234	0.4546	0.5274	0.063*	
C74	0.2651 (3)	0.54342 (15)	0.50045 (9)	0.0466 (6)	
C75	0.3684 (3)	0.56349 (14)	0.46038 (10)	0.0450 (5)	
H75	0.4208	0.6139	0.4617	0.054*	
C76	0.3935 (2)	0.50758 (13)	0.41784 (9)	0.0372 (5)	
N1	−0.00377 (18)	0.44574 (10)	0.27062 (7)	0.0347 (4)	
N2	0.28776 (19)	0.24449 (10)	0.32468 (7)	0.0343 (4)	
O1	0.46323 (17)	0.40792 (11)	0.25980 (6)	0.0502 (4)	
O2	−0.04270 (19)	0.28802 (10)	0.35931 (7)	0.0543 (4)	
Cl1	0.52441 (8)	0.53684 (4)	0.36800 (3)	0.05731 (18)	
Cl2	0.22476 (9)	0.61427 (5)	0.55258 (3)	0.0710 (2)	
Cl3	0.41405 (13)	0.39329 (5)	0.06305 (3)	0.0963 (3)	
Cl4	0.10043 (13)	0.63040 (7)	−0.04898 (3)	0.1064 (3)	

### Atomic displacement parameters (Å^2^)

**Table:**

	*U*^11^	*U*^22^	*U*^33^	*U*^12^	*U*^13^	*U*^23^
C1	0.0365 (12)	0.0663 (16)	0.0561 (15)	0.0121 (11)	0.0088 (11)	0.0120 (13)
C2	0.0388 (12)	0.0477 (13)	0.0374 (12)	0.0057 (10)	0.0034 (9)	0.0070 (10)
C3	0.0359 (11)	0.0340 (11)	0.0335 (11)	−0.0030 (9)	0.0022 (9)	0.0002 (9)
C4	0.0336 (12)	0.0363 (11)	0.0334 (11)	−0.0025 (9)	0.0020 (9)	−0.0024 (9)
C5	0.0314 (10)	0.0298 (10)	0.0292 (10)	−0.0004 (8)	0.0007 (8)	−0.0008 (8)
C6	0.0366 (11)	0.0310 (11)	0.0326 (10)	0.0017 (8)	0.0047 (9)	−0.0002 (9)
C7	0.0332 (11)	0.0348 (11)	0.0305 (10)	−0.0006 (8)	−0.0011 (8)	−0.0009 (9)
C8	0.0420 (12)	0.0352 (11)	0.0303 (10)	0.0008 (9)	−0.0004 (9)	0.0005 (9)
C9	0.0603 (15)	0.0501 (14)	0.0382 (12)	0.0106 (12)	−0.0123 (11)	0.0035 (11)
C10	0.0800 (19)	0.0476 (15)	0.0524 (15)	0.0169 (13)	−0.0087 (13)	0.0122 (12)
C11	0.0717 (17)	0.0365 (13)	0.0612 (16)	0.0109 (12)	−0.0033 (13)	0.0049 (12)
C12	0.0572 (14)	0.0318 (12)	0.0511 (14)	−0.0005 (10)	−0.0007 (11)	−0.0021 (10)
C13	0.0334 (11)	0.0319 (11)	0.0317 (10)	0.0012 (8)	0.0000 (8)	−0.0009 (8)
C14	0.0380 (12)	0.0295 (11)	0.0565 (14)	0.0002 (9)	0.0028 (11)	−0.0023 (10)
C15	0.0382 (13)	0.0392 (13)	0.0774 (17)	0.0008 (10)	−0.0097 (12)	−0.0119 (12)
C16	0.0562 (15)	0.0321 (12)	0.0565 (14)	0.0088 (10)	−0.0228 (12)	−0.0098 (11)
C17	0.0491 (13)	0.0306 (11)	0.0359 (11)	0.0072 (9)	−0.0044 (10)	−0.0025 (9)
C18	0.0714 (17)	0.0435 (14)	0.0425 (13)	0.0094 (12)	0.0064 (12)	−0.0054 (11)
C19	0.124 (3)	0.0498 (16)	0.0408 (15)	0.0223 (17)	0.0108 (16)	−0.0073 (12)
C20	0.135 (3)	0.0565 (18)	0.0467 (16)	0.0218 (19)	−0.0341 (19)	−0.0194 (14)
C21	0.090 (2)	0.0474 (16)	0.0660 (18)	0.0200 (15)	−0.0392 (17)	−0.0195 (14)
C22	0.098 (3)	0.072 (2)	0.126 (3)	0.017 (2)	−0.073 (3)	−0.045 (2)
C23	0.062 (2)	0.088 (3)	0.184 (5)	0.0097 (19)	−0.053 (3)	−0.060 (3)
C24	0.0433 (16)	0.0658 (19)	0.139 (3)	−0.0031 (13)	−0.0139 (17)	−0.034 (2)
C31	0.0401 (12)	0.0392 (12)	0.0371 (11)	−0.0011 (9)	0.0057 (9)	0.0017 (9)
C32	0.0464 (13)	0.0440 (13)	0.0333 (11)	−0.0033 (10)	0.0064 (9)	0.0041 (10)
C33	0.0643 (16)	0.0579 (16)	0.0428 (13)	0.0110 (13)	0.0133 (12)	0.0093 (12)
C34	0.0735 (19)	0.0722 (19)	0.0583 (17)	0.0209 (15)	0.0104 (14)	0.0213 (15)
C35	0.0736 (18)	0.0696 (19)	0.0410 (14)	−0.0047 (15)	−0.0083 (13)	0.0177 (13)
C36	0.098 (2)	0.0576 (17)	0.0318 (13)	−0.0112 (15)	0.0047 (13)	0.0001 (12)
C37	0.0712 (16)	0.0455 (14)	0.0387 (13)	−0.0027 (12)	0.0096 (11)	0.0024 (11)
C71	0.0371 (11)	0.0377 (12)	0.0302 (10)	0.0017 (9)	−0.0060 (9)	−0.0017 (9)
C72	0.0546 (14)	0.0475 (14)	0.0422 (13)	−0.0101 (11)	0.0071 (11)	−0.0067 (11)
C73	0.0588 (15)	0.0581 (16)	0.0423 (13)	−0.0031 (12)	0.0116 (11)	−0.0089 (12)
C74	0.0498 (14)	0.0498 (14)	0.0394 (12)	0.0084 (11)	−0.0071 (10)	−0.0132 (11)
C75	0.0464 (13)	0.0390 (12)	0.0486 (13)	0.0007 (10)	−0.0093 (11)	−0.0077 (10)
C76	0.0364 (11)	0.0374 (12)	0.0372 (11)	0.0030 (9)	−0.0050 (9)	0.0008 (9)
N1	0.0307 (9)	0.0390 (10)	0.0345 (9)	0.0035 (7)	0.0031 (7)	0.0028 (8)
N2	0.0392 (9)	0.0288 (9)	0.0345 (9)	0.0026 (7)	−0.0034 (7)	−0.0013 (7)
O1	0.0323 (9)	0.0762 (12)	0.0424 (9)	0.0019 (8)	0.0045 (7)	0.0069 (8)
O2	0.0505 (10)	0.0524 (10)	0.0617 (11)	−0.0038 (8)	0.0217 (8)	−0.0015 (8)
Cl1	0.0639 (4)	0.0448 (3)	0.0646 (4)	−0.0102 (3)	0.0189 (3)	−0.0010 (3)
Cl2	0.0791 (5)	0.0718 (5)	0.0623 (4)	0.0039 (4)	0.0055 (3)	−0.0346 (4)
Cl3	0.1647 (9)	0.0739 (5)	0.0531 (4)	0.0479 (5)	0.0376 (5)	0.0068 (4)
Cl4	0.1384 (8)	0.1225 (8)	0.0564 (5)	0.0076 (6)	−0.0180 (5)	0.0381 (5)

### Geometric parameters (Å, º)

**Table:**

C1—N1	1.454 (3)	C15—C24	1.374 (4)
C1—H1A	0.9600	C15—C16	1.389 (4)
C1—H1B	0.9600	C16—C21	1.402 (3)
C1—H1C	0.9600	C16—C17	1.408 (3)
C2—N1	1.457 (3)	C17—C18	1.357 (3)
C2—C3	1.510 (3)	C18—C19	1.421 (4)
C2—H2A	0.9700	C18—H18	0.9300
C2—H2B	0.9700	C19—C20	1.361 (5)
C3—C31	1.334 (3)	C19—H19	0.9300
C3—C4	1.500 (3)	C20—C21	1.395 (5)
C4—O1	1.210 (2)	C20—H20	0.9300
C4—C5	1.528 (3)	C21—C22	1.424 (5)
C5—C6	1.528 (3)	C22—C23	1.355 (6)
C5—C7	1.568 (3)	C22—H22	0.9300
C5—C13	1.588 (3)	C23—C24	1.389 (5)
C6—N1	1.448 (3)	C23—H23	0.9300
C6—H6A	0.9700	C24—H24	0.9300
C6—H6B	0.9700	C31—C32	1.457 (3)
C7—C71	1.517 (3)	C31—H31	0.9300
C7—C8	1.522 (3)	C32—C33	1.384 (3)
C7—H7	0.9800	C32—C37	1.393 (3)
C8—N2	1.458 (3)	C33—C34	1.377 (3)
C8—C9	1.511 (3)	C33—H33	0.9300
C8—H8	0.9800	C34—C35	1.376 (4)
C9—C10	1.517 (4)	C34—H34	0.9300
C9—H9A	0.9700	C35—C36	1.362 (4)
C9—H9B	0.9700	C35—Cl4	1.733 (3)
C10—C11	1.514 (4)	C36—C37	1.379 (3)
C10—H10A	0.9700	C36—H36	0.9300
C10—H10B	0.9700	C37—Cl3	1.731 (3)
C11—C12	1.515 (3)	C71—C72	1.387 (3)
C11—H11A	0.9700	C71—C76	1.392 (3)
C11—H11B	0.9700	C72—C73	1.379 (3)
C12—N2	1.457 (3)	C72—H72	0.9300
C12—H12A	0.9700	C73—C74	1.366 (3)
C12—H12B	0.9700	C73—H73	0.9300
C13—N2	1.462 (3)	C74—C75	1.368 (3)
C13—C17	1.525 (3)	C74—Cl2	1.735 (2)
C13—C14	1.578 (3)	C75—C76	1.382 (3)
C14—O2	1.208 (3)	C75—H75	0.9300
C14—C15	1.467 (3)	C76—Cl1	1.733 (2)
			
N1—C1—H1A	109.5	C24—C15—C16	120.5 (3)
N1—C1—H1B	109.5	C24—C15—C14	131.8 (3)
H1A—C1—H1B	109.5	C16—C15—C14	107.7 (2)
N1—C1—H1C	109.5	C15—C16—C21	122.9 (3)
H1A—C1—H1C	109.5	C15—C16—C17	113.7 (2)
H1B—C1—H1C	109.5	C21—C16—C17	123.4 (3)
N1—C2—C3	112.30 (17)	C18—C17—C16	118.8 (2)
N1—C2—H2A	109.1	C18—C17—C13	131.8 (2)
C3—C2—H2A	109.1	C16—C17—C13	108.99 (19)
N1—C2—H2B	109.1	C17—C18—C19	118.5 (3)
C3—C2—H2B	109.1	C17—C18—H18	120.8
H2A—C2—H2B	107.9	C19—C18—H18	120.8
C31—C3—C4	115.66 (18)	C20—C19—C18	122.2 (3)
C31—C3—C2	124.5 (2)	C20—C19—H19	118.9
C4—C3—C2	119.79 (17)	C18—C19—H19	118.9
O1—C4—C3	121.33 (18)	C19—C20—C21	120.8 (3)
O1—C4—C5	121.55 (18)	C19—C20—H20	119.6
C3—C4—C5	117.04 (16)	C21—C20—H20	119.6
C6—C5—C4	108.00 (16)	C20—C21—C16	116.2 (3)
C6—C5—C7	114.36 (15)	C20—C21—C22	128.8 (3)
C4—C5—C7	111.76 (16)	C16—C21—C22	114.9 (3)
C6—C5—C13	111.90 (16)	C23—C22—C21	121.4 (3)
C4—C5—C13	106.54 (15)	C23—C22—H22	119.3
C7—C5—C13	104.03 (15)	C21—C22—H22	119.3
N1—C6—C5	108.20 (15)	C22—C23—C24	122.7 (3)
N1—C6—H6A	110.1	C22—C23—H23	118.7
C5—C6—H6A	110.1	C24—C23—H23	118.7
N1—C6—H6B	110.1	C15—C24—C23	117.6 (3)
C5—C6—H6B	110.1	C15—C24—H24	121.2
H6A—C6—H6B	108.4	C23—C24—H24	121.2
C71—C7—C8	114.53 (16)	C3—C31—C32	129.5 (2)
C71—C7—C5	114.96 (16)	C3—C31—H31	115.2
C8—C7—C5	104.84 (16)	C32—C31—H31	115.2
C71—C7—H7	107.4	C33—C32—C37	116.0 (2)
C8—C7—H7	107.4	C33—C32—C31	123.2 (2)
C5—C7—H7	107.4	C37—C32—C31	120.7 (2)
N2—C8—C9	109.89 (17)	C34—C33—C32	122.9 (2)
N2—C8—C7	102.28 (15)	C34—C33—H33	118.6
C9—C8—C7	116.24 (18)	C32—C33—H33	118.6
N2—C8—H8	109.4	C35—C34—C33	118.5 (3)
C9—C8—H8	109.4	C35—C34—H34	120.8
C7—C8—H8	109.4	C33—C34—H34	120.8
C8—C9—C10	109.7 (2)	C36—C35—C34	121.3 (2)
C8—C9—H9A	109.7	C36—C35—Cl4	119.3 (2)
C10—C9—H9A	109.7	C34—C35—Cl4	119.4 (2)
C8—C9—H9B	109.7	C35—C36—C37	118.9 (2)
C10—C9—H9B	109.7	C35—C36—H36	120.6
H9A—C9—H9B	108.2	C37—C36—H36	120.6
C11—C10—C9	110.7 (2)	C36—C37—C32	122.5 (2)
C11—C10—H10A	109.5	C36—C37—Cl3	117.91 (19)
C9—C10—H10A	109.5	C32—C37—Cl3	119.55 (19)
C11—C10—H10B	109.5	C72—C71—C76	115.28 (19)
C9—C10—H10B	109.5	C72—C71—C7	122.47 (19)
H10A—C10—H10B	108.1	C76—C71—C7	122.25 (18)
C10—C11—C12	110.5 (2)	C73—C72—C71	122.9 (2)
C10—C11—H11A	109.5	C73—C72—H72	118.5
C12—C11—H11A	109.5	C71—C72—H72	118.5
C10—C11—H11B	109.5	C74—C73—C72	119.2 (2)
C12—C11—H11B	109.5	C74—C73—H73	120.4
H11A—C11—H11B	108.1	C72—C73—H73	120.4
N2—C12—C11	109.34 (19)	C73—C74—C75	120.7 (2)
N2—C12—H12A	109.8	C73—C74—Cl2	119.40 (19)
C11—C12—H12A	109.8	C75—C74—Cl2	119.85 (19)
N2—C12—H12B	109.8	C74—C75—C76	118.8 (2)
C11—C12—H12B	109.8	C74—C75—H75	120.6
H12A—C12—H12B	108.3	C76—C75—H75	120.6
N2—C13—C17	109.14 (16)	C75—C76—C71	123.0 (2)
N2—C13—C14	113.25 (16)	C75—C76—Cl1	116.72 (17)
C17—C13—C14	101.40 (16)	C71—C76—Cl1	120.31 (16)
N2—C13—C5	102.07 (15)	C6—N1—C1	112.44 (17)
C17—C13—C5	120.34 (16)	C6—N1—C2	109.68 (16)
C14—C13—C5	111.06 (15)	C1—N1—C2	110.96 (17)
O2—C14—C15	126.7 (2)	C12—N2—C8	113.42 (17)
O2—C14—C13	124.8 (2)	C12—N2—C13	116.49 (17)
C15—C14—C13	108.24 (19)	C8—N2—C13	107.43 (15)
			
N1—C2—C3—C31	153.9 (2)	C17—C18—C19—C20	−0.3 (4)
N1—C2—C3—C4	−23.0 (3)	C18—C19—C20—C21	−1.1 (4)
C31—C3—C4—O1	14.3 (3)	C19—C20—C21—C16	0.6 (4)
C2—C3—C4—O1	−168.5 (2)	C19—C20—C21—C22	−176.1 (3)
C31—C3—C4—C5	−162.48 (18)	C15—C16—C21—C20	−175.8 (2)
C2—C3—C4—C5	14.7 (3)	C17—C16—C21—C20	1.3 (4)
O1—C4—C5—C6	150.4 (2)	C15—C16—C21—C22	1.4 (4)
C3—C4—C5—C6	−32.8 (2)	C17—C16—C21—C22	178.5 (2)
O1—C4—C5—C7	23.8 (3)	C20—C21—C22—C23	176.7 (4)
C3—C4—C5—C7	−159.43 (17)	C16—C21—C22—C23	0.0 (5)
O1—C4—C5—C13	−89.2 (2)	C21—C22—C23—C24	−1.0 (6)
C3—C4—C5—C13	87.6 (2)	C16—C15—C24—C23	0.8 (4)
C4—C5—C6—N1	62.4 (2)	C14—C15—C24—C23	−175.6 (3)
C7—C5—C6—N1	−172.48 (15)	C22—C23—C24—C15	0.6 (6)
C13—C5—C6—N1	−54.5 (2)	C4—C3—C31—C32	179.0 (2)
C6—C5—C7—C71	−12.6 (2)	C2—C3—C31—C32	1.9 (4)
C4—C5—C7—C71	110.51 (19)	C3—C31—C32—C33	38.4 (4)
C13—C5—C7—C71	−134.93 (17)	C3—C31—C32—C37	−146.3 (2)
C6—C5—C7—C8	114.08 (18)	C37—C32—C33—C34	1.2 (4)
C4—C5—C7—C8	−122.83 (17)	C31—C32—C33—C34	176.7 (2)
C13—C5—C7—C8	−8.27 (19)	C32—C33—C34—C35	0.4 (4)
C71—C7—C8—N2	158.36 (16)	C33—C34—C35—C36	−1.4 (5)
C5—C7—C8—N2	31.43 (19)	C33—C34—C35—Cl4	−179.9 (2)
C71—C7—C8—C9	−81.9 (2)	C34—C35—C36—C37	0.8 (4)
C5—C7—C8—C9	151.13 (18)	Cl4—C35—C36—C37	179.3 (2)
N2—C8—C9—C10	−56.6 (2)	C35—C36—C37—C32	0.8 (4)
C7—C8—C9—C10	−172.14 (19)	C35—C36—C37—Cl3	−177.1 (2)
C8—C9—C10—C11	55.6 (3)	C33—C32—C37—C36	−1.8 (4)
C9—C10—C11—C12	−55.5 (3)	C31—C32—C37—C36	−177.4 (2)
C10—C11—C12—N2	55.8 (3)	C33—C32—C37—Cl3	176.12 (19)
C6—C5—C13—N2	−141.69 (16)	C31—C32—C37—Cl3	0.5 (3)
C4—C5—C13—N2	100.49 (17)	C8—C7—C71—C72	−29.0 (3)
C7—C5—C13—N2	−17.73 (18)	C5—C7—C71—C72	92.5 (2)
C6—C5—C13—C17	97.4 (2)	C8—C7—C71—C76	151.57 (19)
C4—C5—C13—C17	−20.4 (2)	C5—C7—C71—C76	−86.9 (2)
C7—C5—C13—C17	−138.64 (17)	C76—C71—C72—C73	1.0 (3)
C6—C5—C13—C14	−20.7 (2)	C7—C71—C72—C73	−178.4 (2)
C4—C5—C13—C14	−138.51 (17)	C71—C72—C73—C74	0.9 (4)
C7—C5—C13—C14	103.27 (18)	C72—C73—C74—C75	−2.2 (4)
N2—C13—C14—O2	56.1 (3)	C72—C73—C74—Cl2	176.50 (19)
C17—C13—C14—O2	172.9 (2)	C73—C74—C75—C76	1.5 (4)
C5—C13—C14—O2	−58.1 (3)	Cl2—C74—C75—C76	−177.16 (17)
N2—C13—C14—C15	−118.41 (19)	C74—C75—C76—C71	0.5 (3)
C17—C13—C14—C15	−1.6 (2)	C74—C75—C76—Cl1	179.48 (17)
C5—C13—C14—C15	127.42 (18)	C72—C71—C76—C75	−1.7 (3)
O2—C14—C15—C24	4.5 (4)	C7—C71—C76—C75	177.69 (19)
C13—C14—C15—C24	178.9 (3)	C72—C71—C76—Cl1	179.33 (17)
O2—C14—C15—C16	−172.1 (2)	C7—C71—C76—Cl1	−1.2 (3)
C13—C14—C15—C16	2.2 (2)	C5—C6—N1—C1	161.08 (17)
C24—C15—C16—C21	−1.8 (4)	C5—C6—N1—C2	−74.95 (19)
C14—C15—C16—C21	175.3 (2)	C3—C2—N1—C6	52.4 (2)
C24—C15—C16—C17	−179.1 (2)	C3—C2—N1—C1	177.25 (19)
C14—C15—C16—C17	−2.0 (3)	C11—C12—N2—C8	−59.1 (2)
C15—C16—C17—C18	174.6 (2)	C11—C12—N2—C13	175.37 (18)
C21—C16—C17—C18	−2.7 (3)	C9—C8—N2—C12	60.0 (2)
C15—C16—C17—C13	0.9 (3)	C7—C8—N2—C12	−175.93 (17)
C21—C16—C17—C13	−176.4 (2)	C9—C8—N2—C13	−169.77 (17)
N2—C13—C17—C18	−52.3 (3)	C7—C8—N2—C13	−45.73 (19)
C14—C13—C17—C18	−172.1 (2)	C17—C13—N2—C12	−63.5 (2)
C5—C13—C17—C18	65.0 (3)	C14—C13—N2—C12	48.6 (2)
N2—C13—C17—C16	120.24 (18)	C5—C13—N2—C12	168.11 (17)
C14—C13—C17—C16	0.5 (2)	C17—C13—N2—C8	168.05 (16)
C5—C13—C17—C16	−122.4 (2)	C14—C13—N2—C8	−79.8 (2)
C16—C17—C18—C19	2.1 (3)	C5—C13—N2—C8	39.66 (18)
C13—C17—C18—C19	174.1 (2)		

### Hydrogen-bond geometry (Å, º)

*Cg*1 is the centroid of the C32–C37 ring.

**Table:**

*D*—H···*A*	*D*—H	H···*A*	*D*···*A*	*D*—H···*A*
C8—H8···O2	0.98	2.50	3.122 (3)	121
C10—H10a···*Cg*1^i^	0.97	2.68	3.5969	158

Symmetry code: (i) −*x*+1, *y*−1/2, −*z*+1/2.
